# Lipopolysaccharide-induced expression of surfactant proteins A_1_ and A_2_ in human renal tubular epithelial cells

**DOI:** 10.1186/1476-9255-10-2

**Published:** 2013-01-12

**Authors:** Jiao Liu, Fengqi Hu, Guirong Wang, Qingshan Zhou, Guohua Ding

**Affiliations:** 1 Department of Medicine, Renmin Hospital of Wuhan University, Wuhan, Hubei 430060, China; 2 Department of Surgery, SUNY Upstate Medical University, Syracuse, New York 13210, American; 3 Division of Nephrology, Renmin Hospital of Wuhan University, Wuhan Hubei 430060, China

**Keywords:** Surfactant protein A_1_, Surfactant protein A_2_, Human renal tubular epithelial cells, Lipopolysaccharide, Inflammatory modulation

## Abstract

**Background:**

Surfactant protein A (SP-A), encoded by two functional genes, SP-A1 and SP-A2, is essential for the inflammatory process and host defence in the lungs. Recent studies have demonstrated the extrapulmonary expression of SP-A. Similar to the lungs, the kidneys are organs exposed to external pathogens. The present study evaluated the expression and location of SP-A in the kidneys. The effect of lipopolysaccharide (LPS) on the expression of SP-A subtypes was also studied in renal tubular epithelial (HK-2) cells.

**Methods:**

Immunohistochemical staining was performed using polyclonal antibody against SP-A. RT-PCR was also performed using mRNA from normal human renal tissues and HK-2 cells. The expressions of the SP-A1 and SP-A2 genes were determined by PCR-based RFLP analysis, gene-specific amplification, and direct sequencing of RT-PCR products. Western blot was conducted to analyse the SP-A protein. HK-2 cells were treated with LPS at various concentrations (0, 0.1, 1, 2, 5, and 10 μg/mL) for 8 h and at 5 μg/mL at various time points (0, 2, 4, 8, 16, and 24 h). The LPS-induced expressions of SP-A1 and SP-A2 mRNA and protein were analysed by RT-PCR and Western blot.

**Results:**

SP-A was localised in the renal tubular epithelial cells in the proximal and distal convoluted tubules. SP-A1 and SP-A2 mRNA and protein were expressed in HK-2 cells and human renal tissues, which were significantly increased in time- and dose-dependent manners after LPS treatment (*P* < 0.05).

**Conclusions:**

Human renal tubular epithelial cells can express both SP-A1 and SP-A2 genes, which may play important roles in the inflammatory modulation of the kidney.

## Background

Pulmonary surfactant, a complex mixture of lipids and proteins, is produced and secreted by alveolar type II cells [1]. Approximately 10% of the surfactant is formed by proteins, which contain the hydrophilic surfactant proteins A and D (SP-A and SP-D, respectively) as well as the hydrophobic surfactant proteins B and C (SP-B and SP-C, respectively) [2]. Human SP-A genes are located on chromosome 10q22-q23 [3]. Two functional genes, SP-A1 and SP-A2, which share 96% and 97% similarities in amino acid and nucleotide sequences, respectively, encode SP-A [4,5]. SP-A also has characteristic structures similar to those of the C-type lectin superfamily, which forms a flower bouquet structure composed of six trimeric subunits, each of which consists of 26 kDa to 35 kDa monomers [1,6]. Alveolar type II and Clara cells in the lungs are the primary cells that secrete SP-A, which plays a role in preventing the pulmonary alveoli from collapsing after expiration [7]. The expression of SP-A, which is not restricted to the respiratory system, was also detected in gastric, intestinal [8], vaginal [9], skin [10], middle ear [11], and Eustachian tube [12] epithelia. Similar to the lungs, the kidneys are derived from the endoderm and are exposed to external pathogens. Thus, the kidney is likely one of the sites where SP-A can be expressed.

SP-A participates in the pathophysiological and physiological regulation of the inflammatory process in the lungs [13]. SP-A can bind to several bacterial, viral, and fungal pathogens, thereby resulting in growth inhibition and opsonisation of these pathogens [14]. SP-A knockout mice were reported to exhibit increased levels of proinflammatory cytokines and decreased ability of phagocytosis by alveolar macrophages [15]. George et al. [16] reported that the complete SP-A gene expression in the lungs is increased after repeated exposure to an inhaled endotoxin. Lipopolysaccharide (LPS), a major component of the outer membrane of Gram-negative bacteria, induces various inflammatory mediators, including tumour necrosis factor, interleukin-1, and interferon [17]. A recent study has also shown that LPS selectively induces SP-A gene expression possibly through TLR2-mediated activation of c-Jun in human alveolar epithelial A549 cells [18]. However, the effect of LPS on the regulation of SP-A expression in human tubular epithelial-like HK-2 cells is not well known.

In the present study, we determined whether SP-A1 and SP-A2 mRNA and proteins are expressed in human renal tissues and HK-2 cells. We also evaluated the effects of LPS on the regulation of SP-A subtype expressions in HK-2 cells.

## Materials and methods

### Materials

Human renal tissue samples obtained from 10 patients (six males and four females, 58 ± 12 years old) who underwent operations for renal cancer were used in the present study. Furthermore, lung tissue samples were obtained from five patients who underwent operations for lung cancer (two females and three males, 62 ± 5 years old). The experimental procedure was carried out according to the protocol approved by the Human Ethics Committee of the Renmin Hospital of Wuhan University in China.

### Cell cultures

Renal tubular epithelial cells (HK-2, a proximal tubular cell line derived from normal kidney; purchased from ATCC, CRL-2190) were cultured at 37°C in a humidified atmosphere of 5% CO_2_ and then treated with Dulbecco’s modified Eagle’s medium (DMEM, Hyclone Pierce, USA) in a mixture of 10% foetal bovine serum (Invitrogen, USA) and 1% ampicillin. The culture medium was changed every 2 d until the cells reached 70% to 80% confluency, at which point they were dissociated with 0.25% trypsin/EDTA. Subsequently, the cells were incubated for 24 and 72 h to determine mRNA and protein expressions, respectively.

### Immunohistochemical experiment

Human renal tissues were embedded in paraffin (4 μm thick) and then immunohistochemically examined using a SP-A polyclonal antibody (supported by Prof. Guirong Wang [19]). After the tissue sections were washed and incubated with 5% H_2_O_2_ in methanol for 30 min at room temperature to inhibit endogenous peroxidase activity, they were incubated with a primary antibody (rabbit anti-human polyclonal SP-A at a dilution of 1:150) at 4°C overnight. The sections were washed in phosphate-buffered saline (PBS) repeatedly and then incubated with an anti-rabbit secondary antibody conjugated with horseradish peroxidase. Negative controls were stained with PBS instead of the primary antibody. Slides were rinsed in PBS and then incubated in diaminobenzidine chromogen (Solarbio, China). Colour development was observed under a microscope. The slides were counterstained with haematoxylin to determine the presence of nuclei.

### RT-PCR

Normal human renal tissues and cultured HK-2 cells were used for RT-PCR analysis of SP-A and GADPH mRNA, and alveolar tissues were used as positive controls. An oligonucleotide dT primer and primer pairs of the target and control genes, respectively, for RT-PCR were purchased from Invitrogen Corporation (Table 1). Total RNA was extracted from each sample with Trizol (Invitrogen, USA) following the manufacturer’s instructions. Total RNA (1 μg) extracted from the tissue was used for the RT reaction (Takara, China), and then 1 μL of cDNA was used for amplification at a final volume of 20 μL according to the supplier’s protocol (Fermentas, Germany). Each PCR product (6 μL) was subjected to electrophoresis on 2% agarose gel.

**Table 1:** Primers used for amplification of SP-A1, SP-A2 and SP-A

**Target Genes**	**Primer Sequences**	**Tm(°C)**	**Cycles**	**Length(bp)**
SP-A	F-GAAGGACGTTTGTGTTGGAA	56.1	35	439
commom to SP-A1 and A2	R-TGGATTCCTTGGGACAGCAA			
SP-A	F-CACTTTTGATGCCATTCAGGAG	56.3	35	295
commom to SP-A1 and A2	R- GGTCAGTCGGGAGTACAGGC			
SP-A1	F-TGGGCAAGGTAATCAGTG	49.1	33	365
	R-GTCCAGGAAGATGGGTTT			
SP-A2	F-TATTGACCGAGCACATACC	49.3	33	212
	R-GTCCAGGAAGATGGGTTT			
GADPH	F-TCCCTGGAGAAGAGCTACGA	53.5	30	200
	R-TCGTCATACTCCTGCTTGCT			

### Detection of the gene expressions of SP-A1 and SP-A2 mRNA

Approximately 10 μL of the PCR product (a primer pair common to SP-A1 and SP-A2, 439 bp) was digested using 2 μL of the restriction enzyme Apa I (Toyobo, Japan), 2 μL of 10× buffer B, and 18 μL of double-distilled water at 37°C for 12 h. The treated PCR product (8 μL) was then electrophoresed on 2% agarose gel.

Another PCR product (a primer pair common to SP-A1 and SP-A2; 295 bp) was subjected to electrophoresis on 2% agarose gel and then purified using a TIANgel Midi purification kit (TianGen, China) according to the manufacturer’s instructions. Sequencing was then directly performed using an ABI 3730xl 96-capillary DNA analyser.

### Western blot

Proteins from human tissues (kidneys and lungs) and HK-2 cells were extracted using RIPA lysate [150 mM sodium chloride, 1.0% Triton X-100, 0.5% sodium deoxycholate, 0.1% sodium dodecyl sulphate (SDS), and 50 mM Tris at pH 8.0]. The total protein concentrations were determined using the bicinchoninic acid protein assay (Hyclone Pierce). Total protein (50 μg) was resolved by reducing 12% SDS-polyacrylamide gel electrophoresis and then transferred electrophoretically at 60 mA onto nitrocellulose membranes at 4°C overnight (Bio-Rad, USA). After the samples were blocked in 5% non-fat milk in Tris-buffered saline, immunoblotting was detected using a primary antibody against SP-A(rabbit anti-human polyclonal SP-A at a dilution of 1:150) and a anti-rabbit secondary antibody conjugated with horseradish peroxidase. Cellular β-actin protein was immunodetected using a human monoclonal antibody against human β-actin (Sigma) as the internal standard. Immunoproducts were detected using enhanced chemiluminescence peroxidase detection reagents (Amersham, Sweden).

### LPS-induced expressions of SP-A1 and SP-A2 mRNA and protein in HK-2 cells

HK-2 cells were incubated with various concentrations (0, 0.1, 1, 2, 5, and 10 μg/mL) of LPS from *Escherichia coli* 0111:B4 (Sigma) for 8 h and then with 5 μg/mL of LPS at different time points (0, 2, 4, 8, 16, and 24 h). The cells were collected to detect the expressions of SP-A1 and SP-A2 mRNA and SP-A protein as described above.

### Statistics

Data were expressed as mean ± SEM. Results were analysed using one-way ANOVA with SPSS 13.0 software. P < 0.05 was considered to indicate statistical significance.

## Results

### Localisation of SP-A in renal tissue

**Figure 1:**
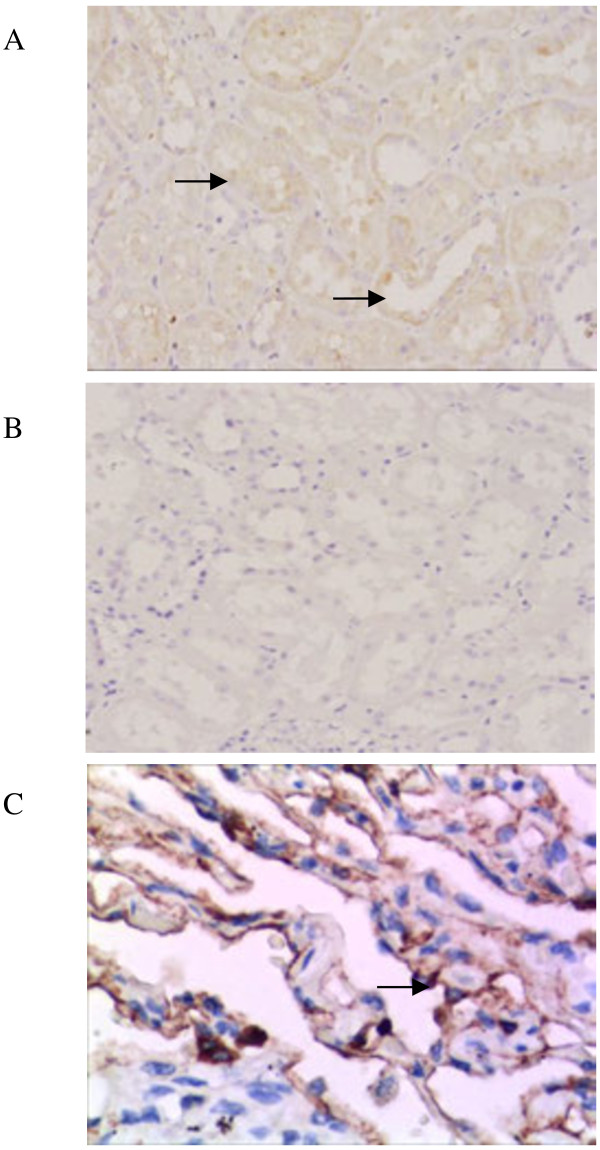
**Immunohistochemical detection of surfactant protein A (SP-A) in renal tissues. (A)** A positive stain was observed in the renal tubular epithelial cells of the proximal and distal convoluted tubules. A polyclonal antibody against human SP-A was used at a dilution of 1:150. **(B)** Negative control sections from human renal tissues, in which phosphate-buffered saline was used instead of the primary antibody. **(C)** Alveolar tissues were used as positive controls. A positive stain was observed in alveolar type II cells. Arrows in the figure point at positive staining for SP-A.

The immunohistochemical experiment using a specific SP-A antibody revealed the localisation of SP-A in the paraffin sections of renal tissues (n = 10). SP-A (Figure 1A) was found in the renal tubular epithelial cells of the proximal and distal convoluted tubules. No significant positive staining was found in the control sections incubated with PBS in place of the primary antibody (Figure 1B).

### Expression of SP-A mRNA in human renal tissues and HK-2 cells

**Figure 2:**
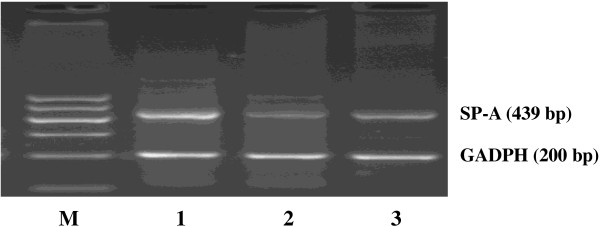
**RT-PCR analysis of the surfactant protein A (SP-A) gene in human tissues.** SP-A and GADPH transcripts were amplified by RT-PCR with gene-specific primer pairs. The products were analysed by 2% agarose gel electrophoresis. Human renal tissues (Lane 2) and HK-2 cells (Lane 3) showed a clear band of SP-A cDNA. PCR amplification products of the lungs were used as positive controls (Lane 1). GAPDH control product was also used (200 bp).

**Figure 3:**
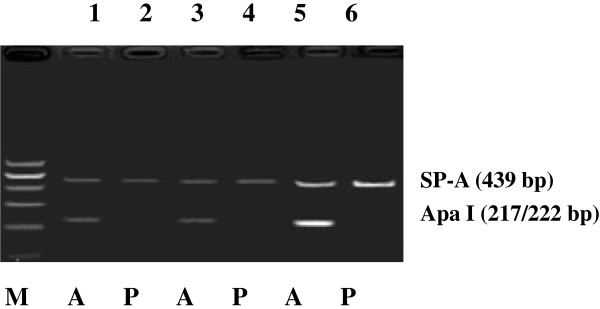
**Surfactant protein A (SP-A) cDNA segments (a mixture of SP-A1 and SP-A2, 439 bp) from renal tissues, HK-2 cells, and the control alveolar tissues were digested by Apa I.** A, SP-A cDNA segments treated with Apa I; P, SP-A cDNA segments untreated with Apa I. SP-A cDNA segments from renal tissues (Lanes 1 and 2) and HK-2 cells (Lanes 3 and 4). SP-A cDNA segments from alveolar tissues were used as positive controls (Lanes 5 and 6).

SP-A-specific cDNA products (a primer pair commom to SP-A1 and SP-A2, 439 bp) were amplified from the renal tissues and HK-2 cells (Figure 2). The sizes of the segment from the renal tissues and HK-2 cells were similar to those from the lungs. However, the band densities were reduced in the renal tissues and HK-2 cells. These findings indicated that the HK-2 cells can transcribe the SP-A genes. The restriction site of the Apa I restriction enzyme only exists in the SP-A1 gene (CTGGGCCC) [5,20]. In the Apa I digestion experiment, the SP-A-specific cDNA segment (a mixture of SP-A1 and SP-A2 cDNA, 439 bp) was digested by Apa I and then subjected to electrophoresis. Electrophoresis of the digested SP-A cDNA of the renal tissues and HK-2 cells revealed two bands at 439 bp and approximately 220 bp. The result was similar to that in the digested SP-A cDNA of alveolar tissues (Figure 3). We performed the Apa I digestion experiment in each sample obtained from 10 patients. All the samples exhibited similar findings without significant differences.

**Figure 4:**
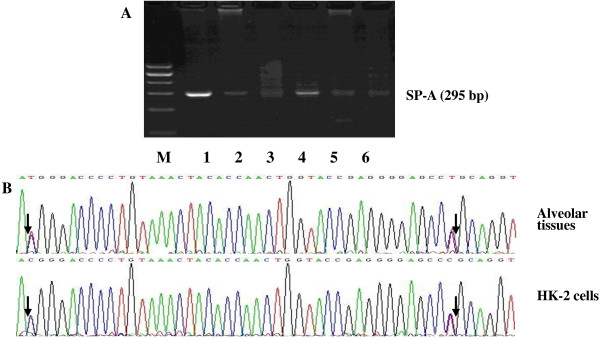
**Sequence data of surfactant protein A (SP-A) cDNA segments (a mixture of SP-A1 and SP-A2, 295 bp). (A)** Gel electrophoresis of SP-A cDNA. Human renal tissues (Lanes 2 and 5) and HK-2 cells (Lanes 3 and 6) showed clear bands of SP-A cDNA. Alveolar tissues were used as positive controls (Lanes 1 and 4). **(B)** DNA sequence. Arrows indicate the nucleotides that differ in SP-A1 and SP-A2 gene sequences. The sequencing result of SP-A cDNA in HK-2 cells revealed the same nucleotide sequence as in alveolar tissue.

**Figure 5:**
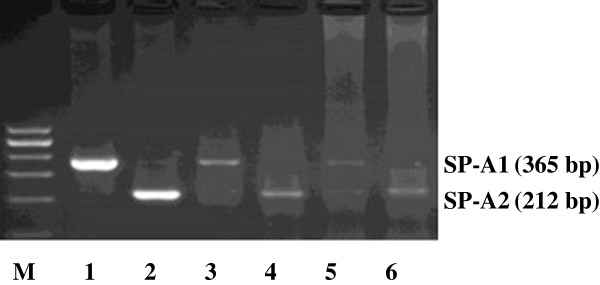
**Specific primer extension analysis for SP-A1 or SP-A2 mRNA transcripts.** SP-A1 (Lane 3) and SP-A2 (Lane 4) mRNA transcripts were detected in human renal tissues. SP-A1 (Lane 5) and SP-A2 (Lane 6) mRNA transcripts were detected in the cultured HK-2 cells. Alveolar tissues were used as positive controls (Lanes 1 and 2). Both SP-A1 and SP-A2 mRNA transcripts were expressed by human renal tissues and cultured HK-2 cells.

Another SP-A-specific cDNA product (another primer pair common to SP-A1 and SP-A2, 295 bp) was analysed through direct sequencing to identify whether SP-A is transcribed from one or both SP-A genes in the HK-2 cells. The sequencing results in Figure 4 show that the 121st and 163rd base sites have two peaks. Each of the two base sites was simultaneously found in two nucleotides (T121G and T163G). Sequence comparison showed that identical transcripts (both SP-A1 and SP-A2) were found in the HK-2 cells and the lungs. We also used the primer pairs specific to SP-A1 or SP-A2. The SP-A1 or SP-A2 cDNA products amplified from the renal tissues and HK-2 cells were the same as those from the positive control alveolar tissues (Figure 5).

### SP-A immunodetection

**Figure 6:**
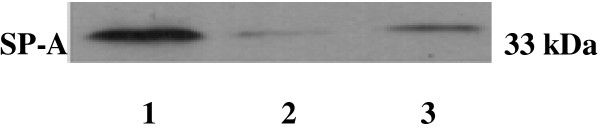
**Western blot analysis of human renal and alveolar tissues and cultured HK-2 cells developed using the surfactant protein A (SP-A) polyclonal antibody.** Immunoblot detection of human renal tissues (Lane 2) and HK-2 cells (Lane 3) showed bands at approximately 33 kDa. Alveolar tissues were used as positive controls (Lane 1). The stained intensity bands of renal tissues and HK-2 cells were decreased compared with those in the lungs.

Western blot analysis of human renal tissues and HK-2 cells showed bands at approximately 33 kDa. The expression levels of SP-A were decreased compared with those in the lungs, as illustrated in Figure 6, in which one out of the 10 samples is shown. As in the HK-2 cells, the experiments were repeated several times.

### LPS-induced expressions of SP-A1 and SP-A2 mRNA and protein in HK-2 cells

**Figure 7:**
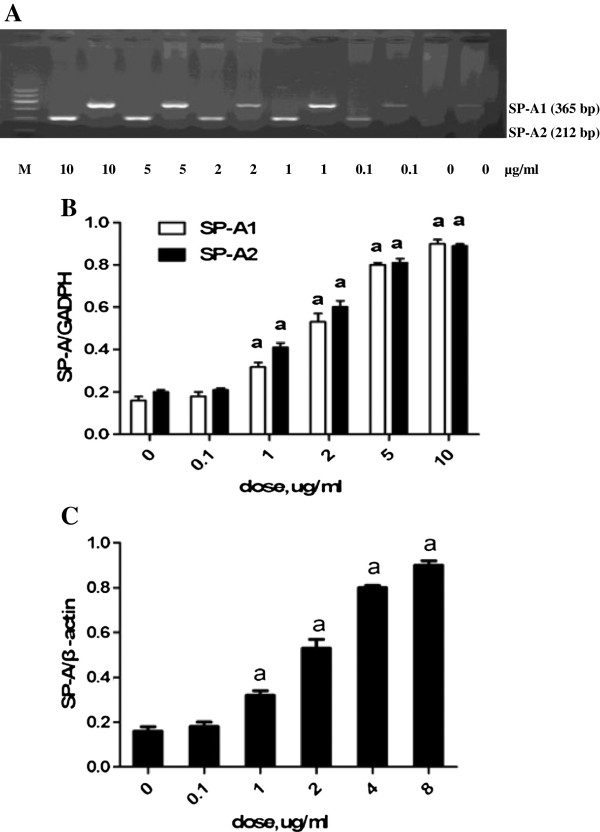
**Dose-dependent effects of lipopolysaccharide (LPS) on the expressions of surfactant protein-A (SP-A) mRNA and protein for 8 h.** HK-2 cells were exposed to 0, 0.1, 1, 2, 5, and 10 μg/mL of LPS for 8 h. (A) Total RNA was prepared for RT-PCR analysis of SP-A mRNA and for gel electrophoresis of SP-A1 and SP-A2 mRNA. (B) Comparison of SP-A1 and SP-A2 mRNA after exposure to different concentrations of LPS for 8 h. (C) Comparison of the SP-A protein after exposure to different concentrations of LPS for 8 h. Each value represents the mean ± SEM for n = 3. The symbol “a” indicates that the value significantly (*P* < 0.05) differed from the control group. M, 100 bp DNA marker.

**Figure 8:**
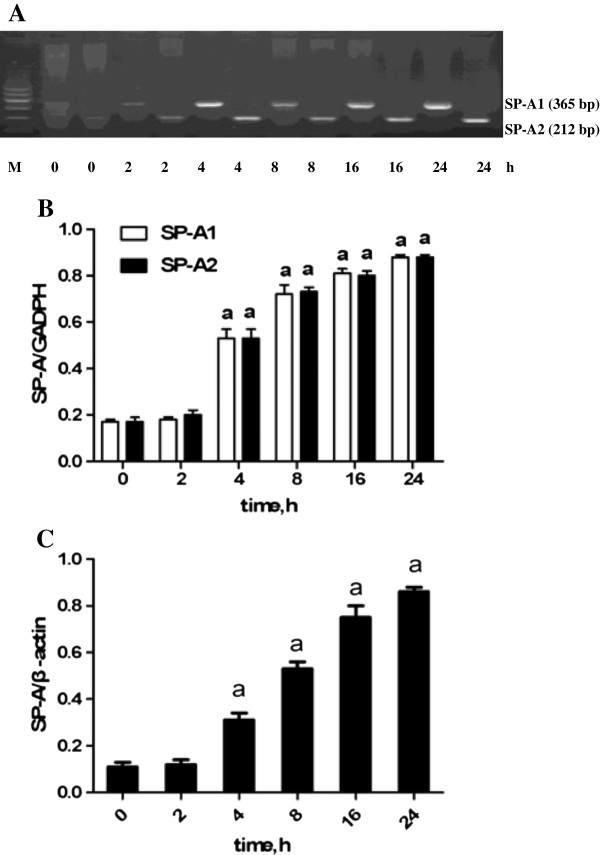
**Time-dependent effects of lipopolysaccharide (LPS) on the expressions of surfactant protein-A (SP-A) mRNA and protein.** HK-2 cells were exposed to 5 μg/mL of LPS for 0, 2, 4, 8, 16, and 24 h. **(A)** Gel electrophoresis of SP-A1 and SP-A2 mRNA. **(B)** Comparison of SP-A1 and SP-A2 mRNA after exposure to 5 μg/mL of LPS at different time points. **(C)** Comparison of SP-A protein after exposure to 5 μg/mL of LPS at different time points. Each value represents the mean ± SEM for n = 3. The symbol “a” indicates that the value significantly (*P* < 0.05) differed from the control group. M, 100 bp DNA marker.

The effects of LPS on SP-A1 and SP-A2 mRNA and protein syntheses were determined by RT-PCR and western blot analysis. In the untreated cells, the expressions of SP-A1 and SP-A2 mRNA and protein were detected (Figure 7). The HK-2 cells exposed to 1, 2, 5, and 10 μg/mL of LPS for 8 h had significantly increased SP-A1 and SP-A2 mRNA and SP-A protein syntheses compared with those exposed to 0 and 0.1 μg/mL of LPS (P < 0.05). The LPS-induced (5 μg/mL) expressions of SP-A1 and SP-A2 mRNA and protein were significantly increased within 2 h (P < 0.05) and maintained beyond 24 h (Figure 8). LPS was found to induce SP-A1 and SP-A2 mRNA and protein syntheses in time- and concentration-dependent manners.

## Discussion

The present study demonstrated the expression and localisation of SP-A within the renal tissue. LPS selectively induced the expressions of SP-A1 and SP-A2 mRNA and protein in human tubular epithelial-like HK-2 cells in time- and dose-dependent manners.

Although the bands for SP-A (33 kDa) in the kidney extracts were detected by Kankavi [21], SP-A mRNA was never detected in the renal tissues of mice [22,23] and humans. The current study presented the exact expressions of SP-A mRNA and protein in human renal tissues. The different results of the present and previous studies may be attributed to the following. First, the present study performed repetitive experiments and collected more samples than previous studies. Second, compared with previous studies, the present study considered different sites of specimen collection in the renal tissues, where SP-A had a higher level of expression. Third, the nucleotide sequence of SP-A in PUBMED has been revised.

Both SP-A1 and SP-A2 transcripts are expressed in adult human alveolar type II cells [24]. McCormick et al. [24] reported that 65% of the SP-A mRNA is encoded by the SP-A2 gene, whereas only 35% is derived from the SP-A1 gene in the lungs. The SP-A1 and SP-A2 genes are differentially expressed in different tissues. Only the SP-A2 gene is predominantly detected in the trachea [25] and submucosal gland cells in the airway [20], whereas SP-A1 and SP-A2 transcript expressions are both detected in the small and large intestines of humans [26]. If the SP-A1 gene is only expressed, then the SP-A cDNA fragment (439 bp) can be digested by Apa I. In this study, the PCR products (439 bp) of the renal tissues and HK-2 cells were digested to 217 and 222 bp bands (bands overlapped at 220 bp). This finding indicated that the SP-A1 gene can be expressed in the renal tissues and HK-2 cells. We also used another primer pair that is common to SP-A1 and SP-A2 (295 bp) to amplify the SP-A cDNA segments of the HK-2 cells and then performed direct sequencing. The sequencing data, which were evaluated by the BLAST tool on PUBMED, correspond to the expected sequences of SP-A1 and SP-A2. The primer pairs specific to SP-A1 or SP-A2 were also used to identify the expressions of the SP-A1 and SP-A2 genes. Considering the result obtained from the Apa I digestion experiment, we conclude that the SP-A1 and SP-A2 genes were expressed in the renal tissues and HK-2 cells in the same way as in the alveolar type II cells. However, the SP-A mRNA level was relatively reduced in the renal tissues and HK-2 cells than in the lungs. We did not determine whether the surface area of the urinary tract is smaller, although the SP-A gene expression in the renal tissue was found to be much lower, which may still be enough to function in local host defence.

In the present study, we have shown that both SP-A1 and SP-A2 mRNA is expressed in renal tissue and the HK-2 cell line. As the SP-A protein can be detected by the polyclonal antibody of SP-A as well in renal tissue and the HK-2 cell line, as shown by western blotting and immunohistochemistry, the polyclonal antibody could potential recognise epitopes in both the SP-A1 and SP-A2 proteins. Therefore, the localisation and synthesis of SP-A occur in the kidneys. Immunostained SP-A is present in the epithelial cells of different tissues, such as in the Eustachian tube [12] and nasal cavities [27]. Immunoreactive SP-A is also found in human Eustachian tube lavage [12] and middle ear fluids [27]. We frequently detected that the renal tubular epithelial cells contain the SP-A protein, which is in good agreement with the findings of a previous study [21].Gram-negative bacteria, such as E. coli, are one of the major causes of urinary tract infection. LPS is the major component of *E*. *coli*. Thus, the severity and time course of kidney inflammation may depend on the amount of LPS. SP-A is the most abundant surfactant protein in the lungs and contributes to lung host defence. A previous study reported that SP-A gene knocked-out mice are susceptible to lung infection caused by several pathogens. In the present study, the levels of SP-A1 and SP-A2 mRNA and protein in the HK-2 cells were significantly increased after LPS treatment. This finding indicated that SP-A may play an anti-inflammatory role in kidney infection.

In summary, the following conclusions are presented. First, human renal tubular epithelial cells can transcribe both SP-A1 and SP-A2 genes. Second, LPS induces SP-A1 and SP-A2 mRNA and protein syntheses in time- and dose-dependent manners. Third, SP-A in the renal tissues may prevent the spread of specific infections from the urinary tract to other body parts. However, the mechanism should be further studied.

## Conclusion

In summary, human renal tubular epithelial cells can express both SP-A1 and SP-A2 genes, which may play important roles in the inflammatory modulation of the kidney.

## Abbreviations

SP-A: Surfactant proteins A; LPS: Lipopolysaccharide; TNF-α: Tumor necrosis factor-α; IL-1: Interleukin-1; DAB: Diaminobenzidine.

## Competing interests

The authors declare that they have no competing interests.

## Authors’ contributions

JL carried out the cell incubation experiment, SP-A1/SP-A2 detection and drafted the manuscript. FQH carried out the SP-A1/SP-A2 expression induced by LPS. GRW participated in the design of the study. QSZ performed the statistical analysis. GHD conceived of the whole study, participated in its design and coordination. All authors read and approved the final manuscript.

## References

[B1] King RJ Clements JA Surface active materials from dog lung Am J Physiol 1972 223 707 714 5068619 10.1152/ajplegacy.1972.223.3.707

[B2] Kuroki Y Voelker DR Pulmonary surfactant proteins J Biol Chem 1994 269 25943 25946 7929300

[B3] Kölble K Lu J Mole SE Kaluz S Reid KB Assignment of the human pulmonary surfactant protein D gene to 10q22-q23 close to the surfactant protein A gene cluster Genomics 1993 17 294 298 10.1006/geno.1993.1324 8406480

[B4] Floros J Steinbrink R Jacobs K Phelps D Kriz R Recny M Sultzman L Jones S Taeusch HW Frank HA Isolation and characterization of cDNA clones for the 35-kDa pulmonary surfactant-associated protein J Biol Chem 1986 261 9029 9033 3755136

[B5] Katyal SL Singh G Locker J Characterization of a second human pulmonary surfactant-associated protein SP-A gene Am J Respir Cell Mol Biol 1992 6 446 452 10.1165/ajrcmb/6.4.446 1372511

[B6] Sano H Kuroki Y The lung collectins, SP-A and SP-D, modulate pulmonary innate immunity Mol Immunol 2005 42 279 287 10.1016/j.molimm.2004.07.014 15589315

[B7] Wong CJ Akiyama J Allen L Hawgood J Localization and developmental expression of surfactant proteins D and A in the respiratory tract of the mouse Pediatr Res 1996 39 930 937 10.1203/00006450-199606000-00002 8725251

[B8] Bourbon JR Chailley-Heu B Surfactant protein in the digestive tract, mesentery, and other organs: evolutionary significance Comp Biochem Physiol A 2001 129 151 161 10.1016/S1095-6433(01)00312-9 11369540

[B9] MacNeill C Umstead TM Phelps DS Lin Z Floros J Shearer DA Weisz J Surfactant protein A, an innate immune factor, is expressed in the vaginal mucosa and is present in vaginal lavage fluid Immunology 2004 111 91 99 10.1111/j.1365-2567.2004.01782.x 14678203 PMC1782386

[B10] Mo YK Kankavi O Masci PP Mellick GD Whitehouse MW Boyle GM Parsons PG Roberts MS Cross SE Surfactant protein expression in human skin: evidence and implications J Invest Dermatol 2007 127 381 386 10.1038/sj.jid.5700561 17008883

[B11] McGuire JF Surfactant in the middle ear and Eustachian tube Int J Pediatr Otorhinolaryngol 2002 66 1 15 10.1016/S0165-5876(02)00203-3 12363416

[B12] Paananen R Glumoff V Sormunen W Voorhout W Hallman M Expression and localization of lung surfactant protein B in Eustachian tube epithelium Am J Physiol Lung Cell Mol Physiol 2001 280 L214 L220 11158999 10.1152/ajplung.2001.280.2.L214

[B13] Mendelson CR Role of transcription factors in fetal lung development and surfactant protein gene expression Annu Rev Physiol 2000 62 875 915 10.1146/annurev.physiol.62.1.875 10845115

[B14] Lawson PR Reid K The roles of surfactant proteins A and D in innate immunity Immunol Rev 2000 173 66 78 10.1034/j.1600-065X.2000.917308.x 10719668

[B15] Linke MJ Harris CE Korfhagen TR McCormack FX Ashbaugh AD Steele P Whitsett JA Walzer PD Immunosuppressed surfactant protein A-deficient mice have increased susceptibility to Pneumocystis carinii infection J Infect Dis 2001 183 943 952 10.1086/319252 11237812

[B16] George CL White ML O'Neill ME Thorne PS Schwartz DA Snyder JM Altered surfactant protein A gene expression and protein metabolism associated with repeat exposure to inhaled endotoxin Am J Physiol Lung Cell Mol Physiol 2003 285 L1337 L1344 12922979 10.1152/ajplung.00064.2003

[B17] Raetz CR Ulevitch RJ Wright SD Sibley CH Ding A Nathan CF Gram- negative endotoxin: an extraordinary lipid with profound effects on eukaryotic signal transduction FASEB J 1991 5 2652 2657 1916089 10.1096/fasebj.5.12.1916089

[B18] Chuang CY Chen TL Chen RM Molecular mechanisms of lipopolysaccharide-caused induction of surfactant protein-A gene expression in human alveolar epithelial A549 cells Toxicol Lett 2009 191 132 139 10.1016/j.toxlet.2009.08.015 19712733

[B19] Luo JM Wan YS Liu ZQ Wang GR Floros J Zhou HH Regularity of distribution of immunoreactive pulmonary surfactant protein A in rat tissues Int J Mol Med 2004 14 343 351 15289884

[B20] Saitoh H Okayama H Shimura S Fushimi T Masuda T Shirato K Surfactant protein A2 gene expression by human airway submucosal gland cells Am J Respir Cell Mol Biol 1998 9 202 209 9698591 10.1165/ajrcmb.19.2.3239

[B21] Kankavi O Immunodetection of surfactant proteins in human organ of Corti, Eustachian tube and kidney Acta Biochim Pol 2003 50 1057 1064 14739994

[B22] Akiyama J Hoffman A Brown C Allen L Edmondson J Poulain F Hawgood S Tissue distribution of surfactant proteins A and D in the mouse J Histochem Cytochem 2002 50 993 996 10.1177/002215540205000713 12070278

[B23] Madsen J Tornoe I Nielsen O Koch C Steinhilber W Holmskov U Expression and localization of lung surfactant protein A in human tissues Am J Respir Cell Mol Biol 2003 29 591 597 10.1165/rcmb.2002-0274OC 12777246

[B24] McCormick SM Boggaram V Mendelson CR Characterization of mRNA transcripts and organization of human SP-A1 and SP-A2 genes Am J Physiol 1994 266 L354 L366 8179012 10.1152/ajplung.1994.266.4.L354

[B25] Khubchandani KR Goss KL Engelhardt JF Snyder JM In situ hybridization of SP-A mRNA in adult human conducting airways Pediatr Pathol Mol Med 2001 20 349 366 11552737

[B26] Lin Z deMello D Phelps DS Koltun WA Page M Floros J Both human SP-A1 and Sp-A2 genes are expressed in small and large intestine Pediatr Pathol Mol Med 2001 20 367 386 11552738

[B27] Kim JK Kim SS Rha KW Kim CH Cho JH Lee CH Lee JG Yoon JH Expression and localization of surfactant proteins in human nasal epithelium Am J Physiol Lung Cell Mol Physiol 2007 292 L879 L884 17209137 10.1152/ajplung.00156.2006

